# An Assessment of Antimicrobial Resistant Disease Threats in Canada

**DOI:** 10.1371/journal.pone.0125155

**Published:** 2015-04-23

**Authors:** Michael J. Garner, Carolee Carson, Erika J. Lingohr, Aamir Fazil, Victoria L. Edge, Jan Trumble Waddell

**Affiliations:** 1 Population Health Assessment and Scenarios Team, Public Health Agency of Canada, Ottawa, Ontario, Canada; 2 Laboratory for Foodborne Zoonoses, Public Health Agency of Canada, Guelph, Ontario, Canada; University of Calgary, CANADA

## Abstract

**Background:**

Antimicrobial resistance (AMR) of infectious agents is a growing concern for public health organizations. Given the complexity of this issue and how widespread the problem has become, resources are often insufficient to address all concerns, thus prioritization of AMR pathogens is essential for the optimal allocation of risk management attention. Since the epidemiology of AMR pathogens differs between countries, country-specific assessments are important for the determination of national priorities.

**Objective:**

To develop a systematic and transparent approach to AMR risk prioritization in Canada.

**Methods:**

Relevant AMR pathogens in Canada were selected through a transparent multi-step consensus process (n=32). Each pathogen was assessed using ten criteria: incidence, mortality, case-fatality, communicability, treatability, clinical impact, public/political attention, ten-year projection of incidence, economic impact, and preventability. For each pathogen, each criterion was assigned a numerical score of 0, 1, or 2, and multiplied by criteria-specific weighting determined through researcher consensus of importance. The scores for each AMR pathogen were summed and ranked by total score, where a higher score indicated greater importance. A sensitivity analysis was conducted to determine the effects of changing the criteria-specific weights.

**Results:**

The AMR pathogen with the highest total weighted score was extended spectrum B-lactamase-producing (ESBL) *Enterobacteriaceae* (score=77). When grouped by percentile, ESBL *Enterobacteriaceae*, *Clostridium difficile*, carbapenem-resistant *Enterobacteriaceae*, and methicillin-resistant *Staphylococcus aureus* were in the 80-100^th^ percentile.

**Conclusion:**

This assessment provides useful information for prioritising public health strategies regarding AMR resistance at the national level in Canada. As the AMR environment and challenges change over time and space, this systematic and transparent approach can be adapted for use by other stakeholders domestically and internationally. Given the complexity of influences, resource availability and multiple stakeholders, regular consideration of AMR activities in the public health realm is essential for appropriate and responsible prioritisation of risk management that optimises the health and security of the population.

## Introduction

Antimicrobial resistance (AMR) of infectious agents is a growing concern to public health organizations. Given how complex and widespread this issue is, effective use of finite resources to improve public health decisions and actions requires prioritization of AMR pathogens. Both the World Health Organization (WHO) and the United States Centers for Disease Control and Prevention (USCDC) have released detailed reports on AMR threats [1, 2], which highlight global and American AMR pathogens of concern, respectively. Since the epidemiology of AMR pathogens differs between countries, national threat assessments are useful to determine country-specific priorities.

Prioritization methodologies continue to inform decision making in public health [2–13]. Unfortunately documentation of the decisions made as a result of the methodology are not as common [3, 8, 9, 12], and only a few describe the methodology in sufficient detail to allow reproduction or adaptation in other settings [3, 10–12]. Recent publications identifying AMR threats were difficult to replicate due to the lack of fully detailed methods for selection of priority pathogens [1, 2]. Providing transparency in the prioritization methodologies enables comparative analyses within and between countries.

The current study seeks to build upon previously published methodologies [2, 3, 14] to develop a systematic and transparent approach to AMR risk prioritization in Canada and presents the results of that process.

## Methods

In 2011, the Robert Koch Institute (RKI) published a methodology that outlined a logical and clear approach to the prioritization of infectious diseases for surveillance and research within Germany [3]. This methodology was the most easily adaptable for our goal of developing a systematic and transparent approach to AMR risk prioritization in Canada. Our methodology is additionally based on previous work developed within the Public Health Agency of Canada (PHAC) that assessed infectious disease risks [14] and the AMR threat assessment work by the USCDC (herein designated the CDC Threat Report) [2]. The risk ranking/prioritization involved several steps: pathogen selection for ranking, criteria selection and definition, weighting of criteria, data capture and pathogen scoring, data quality review, a sensitivity analysis of the weighting of the criteria, and expert review. The data collection and creation of summary figures were conducted in Microsoft Office Excel 2010 [15]. The sensitivity analysis was conducted using D-Sight Software [16].

### Pathogen Selection

Previous work by PHAC identified and ranked 245 infectious disease risks to Canada, of which 138 (56%) exhibited AMR in some capacity [14]. Timeline and information limitations did not allow for assessment of all 138 AMR pathogens. A subset was selected using two criteria: pathogen was ranked in the top 20 infectious disease risks in Canada [14]; or pathogen was outside the top 20, but included in the CDC Threat Report [2]. The resulting list of pathogens was reviewed by authors and other experts in PHAC; additional pathogens of interest to the PHAC for review were added. Enterobacteriaceae were assessed as two groups (carbapenem-resistant and extended spectrum B-lactamase-producing), which included *Klebsiella*, *Escherichia coli* (*E*. *coli*) *and Enterobacter*, rather than as separate pathogens. Other Enterobacteriaceae were evaluated separately (e.g., *Salmonella*, *Shigella*, *Citrobacter*).

### Prioritization Criteria and Scoring

Seven of the 10 criteria (incidence, mortality, case fatality, communicability, treatability, public/political attention, and preventability) were developed based on work completed by PHAC [14], RKI [3], and CDC [2]. The remaining three criteria were based on the CDC Threat Report criteria and cut points were modified for the Canadian context (clinical impact, 10-year projection of incidence, economic impact). Specific definitions for each of the criteria can be found in Table 1.

**Table 1:** Prioritization criteria, scoring definitions, and weights.

Criteria	Definition	Scoring Values			Weight*
		0	1	2	
Incidence	Current Canadian annual incidence of the resistant pathogen	<100 cases per year	100–1000 cases per year	>1000 cases per year	9
Annual Mortality	Current annual Canadian mortality caused by resistant pathogen	<10 deaths per year	10–100 deaths per year	>100 deaths per year	5.25
Case Fatality (%)	Case fatality current associated with the resistant pathogen	<5%	5–25%	>25%	5.25
Communicability	Ability of resistant pathogen to spread between people and cause new infections	There is little to no spread between people	Can spread readily in healthcare settings; person to person spread is rare or uncommon outside healthcare settings	Can spread readily	5.25
Treatability	Availability of effective treatment refers to the availability and effectiveness of antimicrobial agents to treat this resistant pathogen	Medical treatment rarely necessary or treatment successful	One or two classes of alternate antimicrobial agents to treat infections but therapy is usually successful	No effective antimicrobial agents exist to treat infections	7.5
Clinical Impact	Clinical impact is measured by the morbidity or mortality attributable to infection with the resistant pathogen. The clinical impact is based upon the medical consequences of an untreated infection	Infection causes mild disease that may require a visit to the doctor′s office	Infection causes infections that are rarely life-threatening but can require inpatient care	Infection causes life-threatening infections	5.25
Public/Political Attention	Captures the level of public attention and risk perception of resistant pathogens as gauged by media presence, social media and advocacy groups, in addition to the political agenda	Perception of disease risk by general public is low and it is not on political agenda, or disease	Perception of disease risk by general public is moderate, and/or there is political acknowledgement/awareness of the disease	This disease) demands international duties, or the general public′s perception of risk perception is high, or it is explicitly high on political agenda	3
10-year projection of incidence	The 10-year projected incidence of infection with this resistant pathogen if nothing changes (i.e., no new prevention interventions or therapy)	Is unlikely to increase	For diseases that score 1 or 2 on incidence criteria: Up to 2-fold increase. For diseases that score 0 on risk rank incidence criteria:2 to 5-fold	For diseases that score 1 or 2 on incidence criteria: More than 2-fold increase. For diseases that score 0 on risk rank incidence criteria: More than 5-fold	5
Economic Impact	Economic impact encompasses the differential in direct healthcare costs between treatment of a patient with AR infection vs. treatment of a patient with a susceptible infection	No cost to Little (<$500 per case; $1 million for society for all cases)	At least $500 but less than $5000 excess direct costs per case or at least $1 million but less than $10 million for society for all such cases	In excess of $5,000 excess direct costs per case or $10 million or more for society for all such cases	2.5
Preventability	Preventability refers to the availability, effectiveness and extent of implementation of prevention measures that limit the spread of the resistant pathogen	There are no preventive measures	Preventions exist but there are challenges to implementing them	Spread is easily preventable by one or several actors	2

*based on PHAC Working Group consensus

Criteria for each pathogen were researched and scored by one of the five working group members; scoring was on a three point scale (0 = nil or low; 1 = moderate; 2 = high). Each score was verified by a second working group member, with a discussion occurring where scoring diverged. The larger working group came to a consensus decision on remaining issues after the verification stage.

### Data Sources

Where available, PHAC surveillance information (public and internal) was used to score pathogens. When these data were unavailable, scoring was based on information from peer-reviewed and grey literature. To aid interpretation of the results, the data quality was assessed on a four point scale: 1 = Canadian data of high quality; 2 = Canadian data of moderate or poor quality; 3 = non-Canadian data of good quality; and 4 = limited or no good quality non-Canadian data.

### Weighting

Separate from the scoring exercise, weights were assigned to each criterion. Certain criteria contribute more to risk prioritization than others (e.g., incidence of a disease versus work/school absenteeism). To address this, criteria were weighted to reflect the relative importance in the Canadian context. Final weights of the criteria were established by consensus of the working group.

### Ranking of the pathogens

A weighted sum approach was used for ranking. The criterion score was multiplied by the weight assigned to that criterion and summed across all the criteria to arrive at a total risk score, which ranged from 0 (lowest priority) to 100 (highest priority). Prioritized pathogens were also categorized into tiers based on four percentile groupings: those with total risk scores in the 80–100^th^ percentile as the highest priority group (Tier 1); 60–80^th^ medium-high priority (Tier 2); 40–60^th^ medium-low priority (Tier 3); less than 40^th^ percentile low priority (Tier 4). The final ranked list of pathogens was compared with the lists developed by CDC [2] and the WHO list of important pathogens [1].

### Sensitivity analysis

A sensitivity analysis was undertaken to consider other weightings and the impact of data uncertainty. Three different approaches were taken, which included: the impact of an alternative, relatively distinct multi-criteria decision analysis (MCDA) algorithm (a pairwise comparison as opposed to a simple weighted sum approach); the stability of the rankings as a function of a change in the individual criteria weights; and the impact of an alternative set of weights for all the criteria representing an alternative decision maker value set.

#### MCDA Algorithm

The outranking approach [5] is a popular alternative that arrives at a ranking based on using a pairwise comparison. This method tends to be less compensatory than the weighted sum approach and provides a good indication of the sensitivity of the results to the algorithm used.

#### Rank Stability

The stability of the rankings as a function of changing the weights associated with individual criteria was tested using the pairwise comparison algorithm implemented using D-Sight software [15]. The analysis looks at how much of a change in weight a criterion can be exposed to before the resulting rank changes. The first analysis evaluated how much each criterion could be changed before the top ranked pathogen deviated from its ranking. The deviation could be as small as a change of even one position.

#### Alternative value set

The final sensitivity analysis included utilizing a panel of experts that work within the AMR field, and asking them to independently derive weights for the criteria—an exercise that would represent an alternative decision maker value set. This was helpful to see how stable the results are as a function of an entirely new set of criteria weights, as opposed to changing individual criteria weights in sequence as was done in the previous analysis. The panel′s alternative criteria weights put more emphasis on: preventability; economic impact; public attention; clinical impact; communicability; and mortality and less emphasis on: incidence; treatability; and ten year projection.

## Results

**Fig 1:**
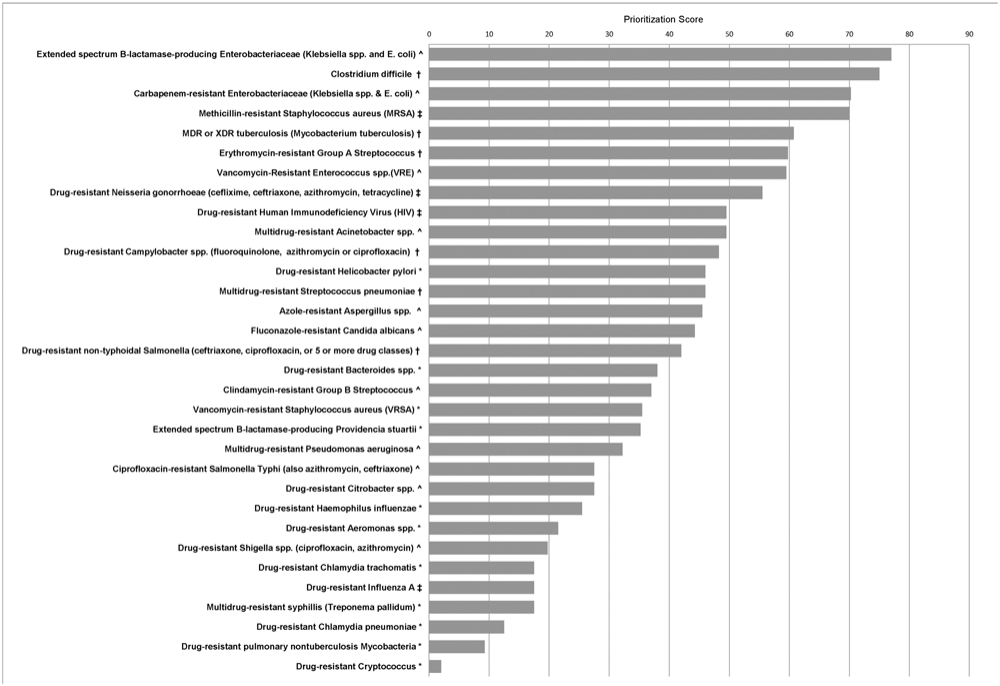
Results from the Public Health Agency of Canada′s prioritization of antimicrobial resistant pathogens. ‡ Canadian data, high quality, disease well characterized and understood in Canadian context, part of national surveillance or disease programs, certainty in scoring. † Canadian data, moderate to poor quality, disease characterized and understood in Canadian context, certainty in scoring. ^ good quality non-Canadian data, disease etiology well understood, moderate confidence in estimates, medium certainty in scoring. * no Canadian data, limited or no good quality non-Canadian data, disease not well understood, uncertainty in scoring.

A total of 32 pathogens were included in the assessment. Table 2 presents the pathogens in four priority groups by their total risk score. The Tier 1 priority group contains 4 pathogens, all with nosocomial relevance (13%); Tier 2 contains 7 (22%) pathogens, Tier 3 and Tier 4 contain ten (31%) and eleven (34%) pathogens, respectively. Fig 1 presents all 32 pathogens by total risk score and data quality category. A comparison of these 32 pathogens with those of the CDC Threat Report and WHO pathogen rankings is found in Table 3. The detailed scores are available in S1 Table (Supplement).

**Table 2:** List of AMR pathogens by priority group, based on total risk score (n = 32) Public Health Agency of Canada.

Tier 1: High Priority group (80–100^th^ percentile)	Tier 2: Medium-high priority group (60 to <80^th^ percentile)	Tier 3: Medium-low priority group (40 to <60^th^ percentile)	Tier 4: Low priority group (<40^th^ percentile)
Extended spectrum B-lactamase-producing Enterobacteriaceae *(Klebsiella* spp., *Enterobacter* and *E*. *coli)* (77)	MDR or XDR tuberculosis (*Mycobacterium tuberculosis*) (61)	Drug-resistant *Helicobacter pylori* (46)	Ciprofloxacin-resistant *Salmonella* Typhi (also azithromycin, ceftriaxone) (28)
*Clostridium difficile (*75)	Erythromycin-resistant Group A *Streptococcus (*60)	Multidrug-resistant *Streptococcus pneumoniae* (46]	Drug-resistant *Citrobacter* spp. (28)
Carbapenem-resistant Enterobacteriaceae *(Klebsiella* spp., *Enterobacter* and *E*. *coli) (*70)	Vancomycin-resistant *Enterococcus* spp.(VRE) (60)	Azole-resistant *Aspergillus* spp. (46)	Drug-resistant *Haemophilus influenzae* (26)
Methicillin-resistant *Staphylococcus aureus* (MRSA) (70)	Drug-resistant *Neisseria gonorrhoeae* (ceflixime, ceftriaxone, azithromycin, tetracycline) (56)	Fluconazole-resistant *Candida albicans* (44)	Drug-resistant *Aeromonas* spp. (22)
	Drug-resistant Human Immunodeficiency Virus (HIV) (50)	Drug-resistant non-typhoidal *Salmonella* (ceftriaxone, ciprofloxacin, or 5 or more drug classes) (42)	Drug-resistant *Shigella* spp. (ciprofloxacin, azithromycin) (20)
	Multidrug-resistant *Acinetobacter* spp. (50)	Drug-resistant *Bacteroides* spp. (38)	Drug-resistant *Chlamydia trachomatis* (18)
	Drug-resistant *Campylobacter* spp. (fluoroquinolone, azithromycin or ciprofloxacin) (48)	Clindamycin-resistant Group B *Streptococcus* (37)	Drug-resistant Influenza A (18)
		Vancomycin-resistant *Staphylococcus aureus* (VRSA) (36)	Multidrug-resistant syphillis (*Treponema pallidum*) (18)
		Extended spectrum B-lactamase-producing *Providencia stuartii* (35)	Drug-resistant *Chlamydia pneumonia* (12)
		Multidrug-resistant *Pseudomonas aeruginosa* (32)	Drug-resistant pulmonary nontuberculosis *Mycobacteria* (9)
			Drug-resistant *Cryptococcus* (2)

**Table 3:** Comparison of pathogens from PHAC Prioritisation, the CDC Threat Report [2] and the WHO bacteria of international concern [1].

Pathogens	2014 PHAC Priority by Tier*	2013 USCDC Threat Report Threat Level	2014 WHO bacteria of international concern
*Clostridium difficile*	**Tier 1**	Urgent	
Carbapenem-resistant Enterobacteriaceae *{Klebsiella*, *Escherichia coli*, *Enterobacter}*	**Tier 1**	Urgent	
Extended spectrum β-lactamase producing Enterobacteriaceae (ESBLs) *{Klebsiella*, *E*. *coli and Enterobacter}*	**Tier 1**	Serious	***E*. *coli*, *K*.*pneumonia*** 3^rd^ gen cephalosporin; resistance conferred by ESBLs, and fluoroquinolone resistant
Methicillin-resistant *Staphylococcus aureus* (MRSA)	**Tier 1**	Serious	***Staphylococcus aureus*** resistant to beta-lactam antibacterial drugs (methicillin, MRSA)
Drug-resistant *Neisseria gonorrhoeae*	**Tier 2**	Urgent	***N*. *gonorrhoeae*** decreased susceptibility to 3^rd^ gen cephalosporins
Multidrug-resistant *Acinetobacter*	**Tier 2**	Serious	
Drug-resistant *Campylobacter*	**Tier 2**	Serious	
Vancomycin-resistant *Enterococcus*	**Tier 2**	Serious	
Drug-resistant *Streptococcus pneumoniae*	**Tier 2**	Serious	***Streptococcus pneumoniae*** penicillin resistant or non-susceptibility (or both)
Drug-resistant tuberculosis	**Tier 2**(MDR, XDR)	Serious	
Erythromycin-resistant Group A *Streptococcus*	**Tier 2**	Concerning	
Vancomycin-resistant *Staphylococcus aureus* (VRSA)	**Tier 3**	Concerning	
Clindamycin-resistant Group B *Streptococcus*	**Tier 3**	Concerning	
Fluconazole-resistant *Candida*	**Tier 3**	Serious	
Multidrug-resistant *Pseudomonas aeruginosa*	**Tier 3**	Serious	
Drug-resistant non-typhoidal *Salmonella*	**Tier 3**	Serious	**Non-typhoidal *Salmonella*** fluoroquinolone resistant
Drug-resistant *Salmonella* Typhi	**Tier 4** Fluoroquinolone, azithromycin, ceftriaxone resistant	Serious	
Drug-resistant *Shigella*	**Tier 4**	Serious	***Shigella*** fluoroquinolone resistant

*Tiers represent percentiles.

**Tier 1: 80–100** ^**th**^;** Tier 2: 60 to <80** ^**th**^;** Tier 3: 40 to <60** ^**th**;^ **Tier 4: <40** ^**th**^

### Sensitivity Analysis

The results of the MCDA and Rank Stability sensitivity analyses were similar, in that top ranked pathogens maintained their positions under different weighting schemes; changes were primarily within the top ten. For example, the Rank Stability analysis showed little movement in ranking even when a maximum weight was assigned to incidence; mortality; clinical impact; ten-year projection; or economic impact. Some criteria: communicability; treatability; public attention; and preventability did have an impact on the top ranked pathogen, though again the change in ranking was observed to be relatively small (from top ranked to second place for instance).

The results of the Alternative Value Set also demonstrated a similar outcome; rankings were stable when looked at in broad categories such as the top 10. Some variation within the broader categories did occur but that was in the order of on a few position changes (e.g., first place to third place, or second place to first place). Overall, the results of the various approaches indicated stability in the findings of the original weighted ranking process.

### Data Quality

A description of the quality of the data sources is provided in Fig 1 and S1 Table. Eleven of the thirty two pathogens were scored using limited non-Canadian data. The majority of the top priority pathogens had data of at least moderate quality upon which to base scoring. Five criteria were based on availability of surveillance/literature data (incidence, mortality, case fatality rate, communicability, treatability). In general, two criteria (clinical impact and economic impact) had little information available across the majority of pathogens. Three criteria (public/political attention, projection of incidence, and preventability) were based on expert opinion informed by the current status of the pathogen and literature.

## Discussion

Two important outcomes resulted from this prioritisation exercise: creation of a Canadian-focused prioritisation methodology and a ranked list of AMR pathogens to help inform thinking around PHAC activities. As Canada establishes AMR priorities, there is a need to develop an approach to understand potential AMR risks for strategic planning. The methods and results should support transparent, science based dialogue between all stakeholders. It is important that the methodology is flexible to changing importance of the criteria, based on decision-maker need and the emergence of new information—both of which are of vital importance when examining AMR pathogens. Moving from the current analysis to finalizing AMR priorities, other considerations such as current public health activities, jurisdictional responsibilities, and context (e.g. vulnerable populations) may need to be accounted for.

In addition to the outcomes themselves (i.e., the assessment tool and ranked list of AMR pathogens), the development of a structured and transparent process is in itself a significant aspect of this project. This is particularly important in situations where there are a broad set of stakeholder groups that do not typically work together as is the case with AMR, which cross cuts the entire field of infectious disease. Equally, though somewhat dependent on time and resources available, stakeholder engagement is critical to the process for input on the criteria used; the weighting assigned to the criteria; and the scoring based on the interpretation of data and evidence reviewed. A decision on when to engage stakeholders should consider adequate time for interactive and meaningful input that will encourage ‘buy-in’ and support for the final results. This needs to be balanced with the feasibility of getting input at different stages and recognising that some stakeholders may desire a more complete product in order to provide comments. The transparency of the current tool allows stakeholders to easily work through the process, allowing them to critique or explore the impact of alternative decisions.

When the ranked AMR pathogens are compared with those of the CDC Threat Report and the WHO list of important pathogens, two of our Tier 1 pathogens, *Clostridium difficile* (*C*. *difficile*) and CRE, were the same as those identified in the top (‘urgent threats’) tier in the CDC Threat Report Report [2]. A third urgent threat identified by the CDC was drug-resistant *Neisseria gonorrhoeae*, which was in our Tier 2. Nine pathogens identified by the WHO as ‘bacteria of international concern’ were all included in our assessment [1]. The WHO list included four of our Tier 1 pathogens (ESBL- and carbapenem-resistant Enterobacteriaceae) but not *C*. *difficile*. Another difference in comparing our findings with those of the WHO was that they separated *E*. *coli* and *Klebsiella pneumoniae*, whereas we grouped them as enterobacteriaceae. The four remaining pathogens highlighted by the WHO (*Neisseria gonorrhoeae*, *Streptococcus pneumoniae*, non-typhoidal *Salmonella*, *Shigella*) were in Tiers 2, 3, 3, and 4, respectively. This might reflect the differences between pathogens of global concern and those of a national concern.

Two pathogens in the assessment, which warrant further discussion, are *C*. *difficile* and *Aspergillus*. *Clostridium difficile* poses a slightly different risk than the other pathogens considered. In general, *C*. *difficile* infections are not resistant to the antimicrobials used to treat them, however cases may be related to antimicrobial use, and as such are useful to include as an AMR threat in this situation [2,17,18]. There are strains of *C*. *difficile* with AMR, but our evaluation was based on the pathogen itself, rather than the resistant strain; whereas for the other pathogens, the evaluation was completed for the resistant strain(s).

Expanding the assessment to include pathogens of potential future importance allows proactive assessment to reduce potential risk from these pathogens in a Canadian context. As an example of this, the assessment process identified the emergence of drug-resistant *Aspergillus* as a potential pathogen of concern in Canada. The European Centre for Disease Prevention and Control performed a risk assessment on the spread of azole-resistant *Aspergillus* [19] and found a mortality rate of invasive azole-resistant *A*. *fumigatus* approaching 90%. Currently the resistant species have not been found in Canada; however actions to mitigate its arrival may be useful for decision makers to consider.

The use of reference ranges in categorical scoring of the criteria allowed practical and useful distinctions to be made based on readily available information. For example, categorizing incidence as <100, 100–1000, and >1000 cases per year, still facilitates decision-making in the face of potentially imprecise data. A strength of the methodology and process is that the entire exercise from the criteria selection, data gathering, and analysis took approximately two months by five people dedicated to the project on a part-time basis. The relatively low resource requirements and simplicity of the method allows for repetition, which can be on a scheduled or ongoing basis, allowing for updates to accommodate integration of new and emerging AMR pathogens, better quality or new data, an expanded list of pathogens, and changing perceptions of risk. Future iterations of this analysis may require revision of the criteria to better suit the unique needs of the risk managers or policy-makers. Finally, the simplicity of the method allows for policy-makers and non-experts to understand what was done, resulting in broader engagement and accessibility and appears to increase buy in, support and use of the findings.

### Limitations

Given the number of pathogens that were scored using poor non-Canadian data (11 of 32) the assessment of those pathogens may not reflect the current Canadian situation as accurately as the situation for the pathogens with good data. The data quality and information gaps for certain pathogens required use of expert opinion to inform some of the scores. Given the unavoidable paucity of surveillance information and data for some pathogens in Canada, this was considered to be the best way forward; data availability for each score is provided in S1 Table and allows for open critique of those opinions. There are additional criteria and factors that were not included that are worth considering during the application of the results, especially when attempting to address a specific policy question. These include, but are not limited to: evaluation of different antimicrobials that the pathogens are resistant to; premature mortality (i.e., whether the resistant pathogen primarily impacts the young or old); vulnerable populations (i.e., drug-resistant TB occurs more within immigrant populations); gaps in information, and the breadth of the assessment. Different groupings of bacteria (within families or separately) to evaluate pathogens with similar resistance genes could also be considered (i.e., alternate ways of grouping the Enterobacteriaceae which readily share resistance genes, such as carbapenem resistance genes). While, the current method is pathogen-specific; there are alternate ways of framing AMR to inform action and decision-making. Combining AMR threat assessment with initiatives to increase prudent use of antimicrobials are likely to result in greater mitigation than focussing on the pathogen threat alone.

## Conclusion

Given the large number of pathogens with resistance, a systematic transparent method for prioritization is essential. While there are many other criteria and considerations (health, economic, social) for prioritization, the list of AMR pathogens presented here can form a basis for AMR surveillance and research priorities, and the targeting of intervention efforts. This work has already served to inform the list of pathogens that are being prioritized for surveillance in the Agency. The application of this method by other groups and in different jurisdictions would allow an exploration of which considerations and criteria for prioritization vary depending on the context, and which ones are constant. We should not limit our future priority setting activities related to AMR by what we currently do, rather we should always endeavour to regularly reconsider a broad list of AMR pathogens to inform our decision making processes in protecting the health of our populations. It is our hope that this work will serve as an additional example to decision-making bodies about the feasibility and importance of transparent and systematic priority setting.

## Supporting Information

S1 Table AMR pathogens prioritization—Results of pathogen scoring. (XLSX)
